# Predicting outcomes at the individual patient level: what is the best method?

**DOI:** 10.1136/bmjment-2023-300701

**Published:** 2023-06-14

**Authors:** Qiang Liu, Edoardo Giuseppe Ostinelli, Franco De Crescenzo, Zhenpeng Li, Anneka Tomlinson, Georgia Salanti, Andrea Cipriani, Orestis Efthimiou

**Affiliations:** 1 Department of Psychiatry, University of Oxford, Oxford, UK; 2 Oxford Precision Psychiatry Lab, NIHR Oxford Health Biomedical Research Centre, Oxford, UK; 3 Department of Engineering Mathematics, University of Bristol, Bristol, UK; 4 Oxford Health NHS Foundation Trust, Warneford Hospital, Oxford, UK; 5 Institute of Social and Preventive Medicine (ISPM), University of Bern, Bern, Switzerland; 6 Institute of Primary Health Care (BIHAM), University of Bern, Bern, Switzerland

**Keywords:** Depression & mood disorders

## Abstract

**Objective:**

When developing prediction models, researchers commonly employ a single model which uses all the available data (*end-to-end* approach). Alternatively, a *similarity-based* approach has been previously proposed, in which patients with similar clinical characteristics are first grouped into clusters, then prediction models are developed within each cluster. The potential advantage of the similarity-based approach is that it may better address heterogeneity in patient characteristics. However, it remains unclear whether it improves the overall predictive performance. We illustrate the similarity-based approach using data from people with depression and empirically compare its performance with the end-to-end approach.

**Methods:**

We used primary care data collected in general practices in the UK. Using 31 predefined baseline variables, we aimed to predict the severity of depressive symptoms, measured by Patient Health Questionnaire-9, 60 days after initiation of antidepressant treatment. Following the similarity-based approach, we used *k*-means to cluster patients based on their baseline characteristics. We derived the optimal number of clusters using the Silhouette coefficient. We used ridge regression to build prediction models in both approaches. To compare the models’ performance, we calculated the mean absolute error (MAE) and the coefficient of determination (R^2^) using bootstrapping.

**Results:**

We analysed data from 16 384 patients. The end-to-end approach resulted in an MAE of 4.64 and R^2^ of 0.20. The best-performing similarity-based model was for four clusters, with MAE of 4.65 and R^2^ of 0.19.

**Conclusions:**

The end-to-end and the similarity-based model yielded comparable performance. Due to its simplicity, the end-to-end approach can be favoured when using demographic and clinical data to build prediction models on pharmacological treatments for depression.

## Introduction

Clinical prediction models aim to predict healthcare outcomes based on a set of baseline variables (predictors).^1^ Accurate prediction models may be used to facilitate clinicians’ decisions on screening and treatment,^2^ and may therefore lead to optimising clinical care.^3^ However, real-world patient data are often heterogeneous and noisy, which may reduce the ability to predict clinical outcomes and disease trajectories.^4^ This problem is especially significant in mental health research.^5^

A commonly applied approach to prediction modelling is the ‘end-to-end’ approach, where a (statistical or machine learning) model is trained on all available data at once to explore linear or non-linear relationships between predictors and outcomes.^6^ An alternative approach is to adopt a two-stage method. In the first stage, an unsupervised algorithm groups patients into *homogeneous* clusters according to similar baseline characteristics, that is, using only patient predictors but not outcomes.^7^ In the second stage, a separate prediction model is developed within each cluster, to provide patient-level predictions of the outcome of interest.^8^ The idea behind this approach is that we try to better capture the heterogeneity of patient characteristics by bypassing the need to create a single, complex model, valid for all possible patient subgroups and types. The two-stage method has previously yielded improved predictive performance compared with the usual end-to-end approach in some applications, such as for predicting mortality and readmission in patients with acute myocardial infarction and for diagnosis and outcome prediction in patients with kidney diseases.^9 10^ It remains however unclear whether applying this method in other settings, such as in mental health outcomes prediction, may also lead to improved performance.

In this paper, we aimed to compare the performance of end-to-end and similarity-based approaches in predicting depression severity using a large data set of patients with depression.

## Methods

### Study design and patients

Data were obtained from the QResearch primary care research database (https://www.qresearch.org/), which contains anonymised electronic healthcare records of over 35 million patients registered with 1500 general practices in the UK. We initially identified an open cohort of patients with depression aged >18 years at the study entry date, drawn from patients registered with eligible practices since 1 January 1998. We wanted to focus on the case of predicting an absolute outcome rather than the relative effects between treatments so we included patients with depression who were prescribed fluoxetine, as it was the first selective serotonin reuptake inhibitor approved by regulatory agencies internationally and has since been the most used, active comparator in antidepressant clinical trials.^11^ We excluded patients who had a previous episode of depression in the year before or a previous prescription of antidepressants in the year before. We also excluded patients who were prescribed two or more antidepressants at the same time, a mood stabiliser or an antipsychotic (see online supplemental file for further information).

10.1136/bmjment-2023-300701.supp1 Supplementary data

We aimed to predict the severity of depressive symptoms measured after 60 days from the initiation of antidepressant treatment.^12^ Depressive symptom severity was measured using the Patient Health Questionnaire-9 (PHQ-9), a validated self-rating scale.^13^ If a patient did not have a depression score recorded after 60 days, we considered at least 21 days after the diagnosis and up to 90 days after as valid measurement.^12^ If there were two or more eligible depression scores, we chose the one closest to our target time point (ie, 60 days after the initiation of antidepressant treatment). If depressive symptoms were assessed using a different questionnaire, we transformed other depression scales, such as Hospital Anxiety and Depression Scale and Beck’s Depression Inventory II, into PHQ-9 following a previously validated algorithm.^14^ Only patients with a post-treatment depression score were included in the analysis.

Based on a recent literature review,^15^ we selected 31 candidate predictors, including baseline characteristics such as demographic variables, condition-specific variables (eg, depression severity) and information relevant to previous treatments and comorbid conditions. A detailed list of these predictors can be found in the online supplemental file. We also provide a mock data set of five patients in online supplemental table S1 to help readers understand the structure of our data.

### Multiple imputations for missing data

There was missing information for several predictors, with approximately 1% of the data missing for smoking status and Townsend deprivation score, 13% for ethnicity and body mass index, and 22% for the baseline PHQ-9 score. We did not exclude subjects with missing predictors as this would have greatly reduced the sample study size, thus decreasing precision and power. Instead, we deployed a multiple imputation method,^16^ where we used additive regressions to impute missing values. We generated 10 imputed data sets.^17^ We did not use the imputed PHQ-9 outcomes for model development or evaluation.

### End-to-end approach

Following the end-to-end approach, a model would use the whole training data set and take 31 predictors as input to predict the PHQ-9 score at 60 days. Many prediction models can be used (eg, statistical models such as simple linear regression, or any type of machine learning model, such as random forest or multilayer perceptron). However, using the same data set, we previously found no evidence of increased performance when using advanced machine learning methods.^18^ Therefore, in this paper we used a linear regression model with L2 regularisation (ridge regression). To find the optimal hyperparameter, that is, the penalty of the ridge regression model, we used a 10-fold cross-validation method, as detailed in the online supplemental file.

We fitted the model separately on each of the 10 imputed data sets. To make a prediction for a new patient, we used the 31 predictors as input in each of the 10 models and averaged their predictions as the final output.

### Similarity-based approach

In the similarity-based approach, in the first stage, we grouped patients with similar clinical characteristics into several clusters. Specifically, we used the *k*-means algorithm to split the data set into *k* clusters (see below how *k* was determined). We chose this algorithm because of its simplicity and ease of interpretation, which contributed to it being one of the most popular clustering algorithms in the medical domain.^19^ Before fitting the *k*-means algorithm, for categorical predictors we used one-hot encoding, that is, we converted each categorical predictor into a series of binary ones.^20^ We standardised the predictors before passing them on to *k*-means algorithm.

Regarding the second stage, to be able to make a fair comparison between the two approaches, the model architecture of the end-to-end approach should be identical to the one used within each cluster in the similarity-based approach. Thus, at the second stage, that is, after identifying the clusters, we applied the same modelling strategy as in the end-to-end approach (ie, a unique ridge regression model within each cluster).

Finally, we ended up with 10 sets of models (one set per imputed data set) where each set contained one clustering model and *k* ridge models. To predict a new individual, we used each of these sets of models to first identify the cluster that the patient belonged to according to his/her baseline characteristics and then used the corresponding cluster-specific model to make predictions. We averaged these 10 predictions (one from each multiple imputed data set) to obtain the final ‘overall’ prediction.

The number of clusters (*k*) can have a big impact on the clustering procedure of the first stage described above. In order to choose the optimal *k*, we used the Silhouette coefficient, which measures the goodness of fit of the *k*-means algorithm.^21^ A detailed explanation of the Silhouette coefficient can be found in the online supplemental file. The Silhouette coefficient ranges from −1 to 1, where 1 means that clusters are perfectly separated and can be very clearly distinguished. Values near 0 indicate overlapping clusters, with no clear distinctions between them. Negative values generally indicate clusters are assigned incorrectly, that is, a different cluster is more similar to the one each sample is assigned into.

Here we explored values of *k* in (2, 3, …, 8). Following previous research,^22^ for each *k* we fitted the k -means algorithm on 1000 bootstrap samples (100 per multiple imputed data set) and calculated the Silhouette coefficient. The optimal number of clusters was determined as the value of k leading to the highest coefficient.

To assess the sensitivity of the results to the choice of the optimal number of clusters, we varied *k* from 2 to 8 and measured the mean absolute error (MAE) and the coefficient of determination (R^2^) using the same bootstrap method detailed above.

### Evaluation of model performance via internal validation

We used R^2^ to evaluate model performance on the PHQ-9 score prediction. R^2^ is scale-free and measures the proportion of the total variation of the PHQ-9 score explained by a model. It is one of the most common measures of model performance for continuous outcomes, such as the one analysed in our paper. Furthermore, following recommendations, for example by Poldrack *et al*,^23^ we additionally used MAE. MAE measures the average magnitude of errors in pairs of observed and predicted outcomes. A lower MAE indicates better model performance.

We calculated the performance metrics using bootstrapping to correct for optimism. For each imputed data set, we first resampled data with a random seed and created a bootstrap sample as the training set. We then developed the end-to-end and similarity-based models in the bootstrap sample. We used out-of-sample patients, that is, patients who were not included in the bootstrap sample, to make predictions using the developed models. By construction, each bootstrap sample had the same size as the original data set and contained approximately 70% of the patients in the original data set (some patients were duplicated), leaving approximately 30% of the patients for testing. Next, we calculated the MAE and R^2^ for both approaches. We then changed the random seed and performed bootstrapping 100 times per imputed data set (1000 times in total). In the end, we averaged the 1000 values of each metric and used the 2.5th–97.5th percentile to obtain their CIs.

### Implementation details

The analyses were conducted on a computer with an Intel Xeon Gold 6246 12-Core central processing unit. Data preparation and cleaning were conducted in Stata V.16.1.^24^ Multiple imputation was carried out using Hmisc in R.^25^ Clustering, model fitting and evaluation were conducted in Python V.3.8.^26^ We provide online demo code for deploying both approaches using self-programmed routines available at https://github.com/oceanlq/Samplecodeforpublication/blob/main/endtoendvspatientsimilarity-basedpredictiveapproach.ipynb.

## Results

We identified a total of 16 384 patients meeting our inclusion criteria. A description of the clinical and demographic characteristics of these patients is shown in table 1.

**Table 1:** Descriptive statistics of the study cohort for analysis of severity of depression symptoms (N=16 384)

Characteristics	Sample n (%)/mean (SD)	Missing n (%)
Predictors: demographic		
Sex		0
Female	10 226 (62.41)	
Male	6158 (37.59)	
Age	41.99 (14.20)	0
Body mass index	27.34 (6.41)	2184 (13.33)
Smoking		236 (1.44)
Yes	4944 (30.18)	
No	11 204 (68.38)	
Ethnic group		2438 (14.88)
White	12 834 (78.33)	
African/Caribbean	223 (1.36)	
Asian	544 (3.32)	
Other	345 (2.11)	
Townsend deprivation score		38 (0.23)
1 (least deprived)	3816 (23.29)	
2	3923 (23.94)	
3	3538 (21.59)	
4	2935 (17.91)	
5 (most deprived)	2134 (13.02)	
Predictors: depression-specific		
Baseline PHQ-9 score	17.45 (4.74)	3630 (22.16)
First episode		0
Yes	11 018 (67.25)	
No	5366 (32.75)	
Previous antidepressant use		0
Any antidepressant	6290 (38.39)	
Any SSRI	5196 (31.71)	
Fluoxetine	3781 (23.08)	
Previous psychotherapy	151 (0.92)	0
Previous referral to secondary care	56 (0.34)	0
Childhood maltreatment	24 (0.15)	0
Predictors: comorbid conditions at baseline		
Coronary heart disease	423 (2.58)	0
Stroke	253 (1.54)	0
Diabetes	827 (5.05)	0
Epilepsy	186 (1.14)	0
Hypothyroidism	627 (3.83)	0
Arthritis	1188 (7.25)	0
Anxiety	2623 (16.01)	0
Migraine	1529 (9.33)	0
Predictors: use of other drugs at baseline		
Antihypertensive	1426 (8.70)	0
Aspirin	593 (3.62)	0
Statins	1327 (8.10)	0
Anticoagulants	106 (0.65)	0
Non-steroidal anti-inflammatory drugs	632 (3.86)	0
Anticonvulsants	219 (1.34)	0
Hypnotics	1214 (7.41)	0
Bisphosphonates	44 (0.27)	0
Contraceptives	1026 (6.26)	0
Outcome		
PHQ-9 score at 2 months	11.70 (6.34)	0

n, number; PHQ-9, Patient Health Questionnaire-9; SSRI, selective serotonin reuptake inhibitor.

### Performance of the end-to-end and similarity-based approaches

**Figure 1:**
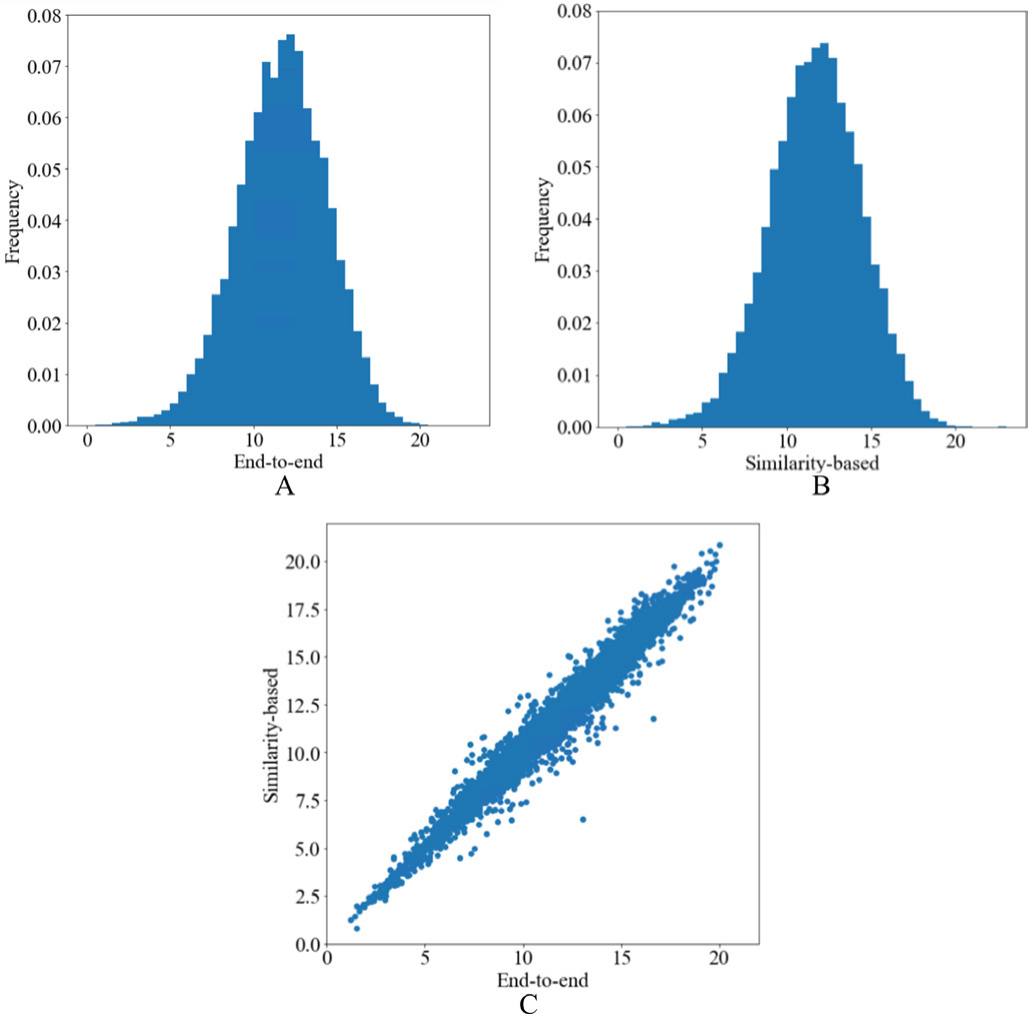
Histograms of predicted Patient Health Questionnaire (PHQ-9) scores of (A) the end-to-end approach and (B) the similarity-based approach (*k*=4), and (C) the scatter plot of predictions of both approaches.

Overall, we found modest to low predictive performance for both approaches. Predictions were similar between the two approaches (see figure 1 and online supplemental figure S1 for a comparison of predicted PHQ-9 scores and table 2 for full details).

**Table 2:** Evaluation of post-treatment Patient Health Questionnaire (PHQ-9) predictions made by the end-to-end and similarity-based approaches

	Number of clusters, *k*	MAE (95% CI)	R^2^ (95% CI)
End-to-end (apparent)	NA	4.56	0.23
End-to-end (internal validation)	NA	4.64 (4.57, 4.71)	0.20 (0.18, 0.21)
Similarity-based (apparent)	4	4.55	0.23
Similarity-based (internal validation)	Optimal *k* for each bootstrap sample	4.65 (4.58, 4.72)	0.19 (0.18, 0.21)
Similarity-based (sensitivity analysis)	2	4.67 (4.60, 4.74)	0.19 (0.17, 0.20)
	3	4.66 (4.59, 4.73)	0.19 (0.17, 0.21)
	5	4.68 (4.61, 4.75)	0.18 (0.17, 0.20)
	6	4.68 (4.61, 4.75)	0.18 (0.16, 0.20)
	7	4.69 (4.62, 4.76)	0.18 (0.16, 0.20)
	8	4.70 (4.63, 4.77)	0.17 (0.15, 0.19)

CI is calculated as the 2.5th–97.5th percentile of bootstrap estimates.

MAE, mean absolute error; NA, not applicable; R^2^, coefficient of determination.

The apparent predictive performance of the end-to-end approach was 4.56 for MAE and 0.23 for R^2^. The performance after internal validation was only slightly worse (MAE=4.64, R^2^=0.20), indicating only minimal overfitting. We provide the histogram of predicted PHQ-9 scores from the end-to-end model in figure 1A. The average coefficients of the ridge end-to-end models developed on 10 imputed data sets can be found in online supplemental table S2. The similarity-based approach achieved comparable performances with the end-to-end approach both in apparent performance metrics (MAE=4.55, R^2^=0.23) and in the internally validated ones (MAE=4.65, R^2^=0.19).

**Figure 2:**
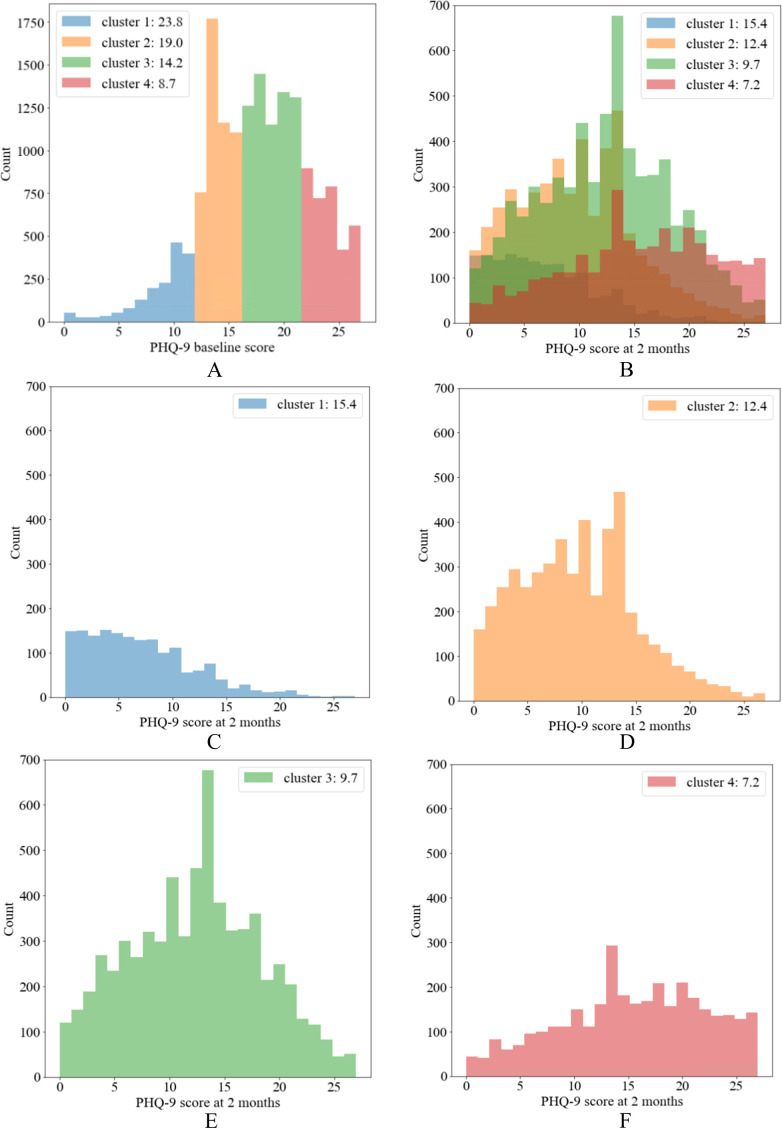
Histograms of Patient Health Questionnaire (PHQ-9) scores in the four identified clusters of patients. 'Values in the legends are the mean PHQ-9 scores. (A) Observed PHQ-9 scores at baseline, (B) observed PHQ-9 scores at 2 months and (C–F) observed PHQ-9 scores at 2 months for each cluster.

In the first stage of the similarity-based model, we found that *k*=4 had the highest average Silhouette coefficient across all imputed data sets (online supplemental table S3, calculated based on 1000 bootstrap samples). The average Silhouette coefficient was 0.15, suggesting extensive overlap between the clusters. In online supplemental table S4, we reported the patients’ characteristics of the clusters identified with the similarity-based approach using the optimal number of clusters (*k*=4) for one randomly selected imputed data set. To facilitate the presentation of results, we named clusters from 1 to 4 following the average baseline PHQ-9 scores of each cluster (ie, highest average baseline score in cluster 1 and lowest in cluster 4). The observed PHQ-9 scores are shown in figure 2. In addition to baseline PHQ-9 score, the clusters also showed a directionality (ie, increasing or decreasing from cluster 1 to 4) for other patient characteristics. For example, patients in cluster 1 were predominantly smokers, younger, white, had higher Townsend deprivation scores (higher level of material deprivation), and had a lower level of comorbidity with coronary heart disease, stroke/transient ischaemic attacks and chronic inflammatory diseases as compared with other clusters. Cluster 1 was also associated with lower use of antihypertensive drugs, aspirin, statins and anticoagulants compared with the other clusters. On the contrary, the use of non-steroidal anti-inflammatory drugs and hypnotics was more frequent in cluster 1. We repeated the clustering procedure multiple times and found that the choice of the multiple imputed data sets did not materially change our findings and the clustering results.

**Figure 3:**
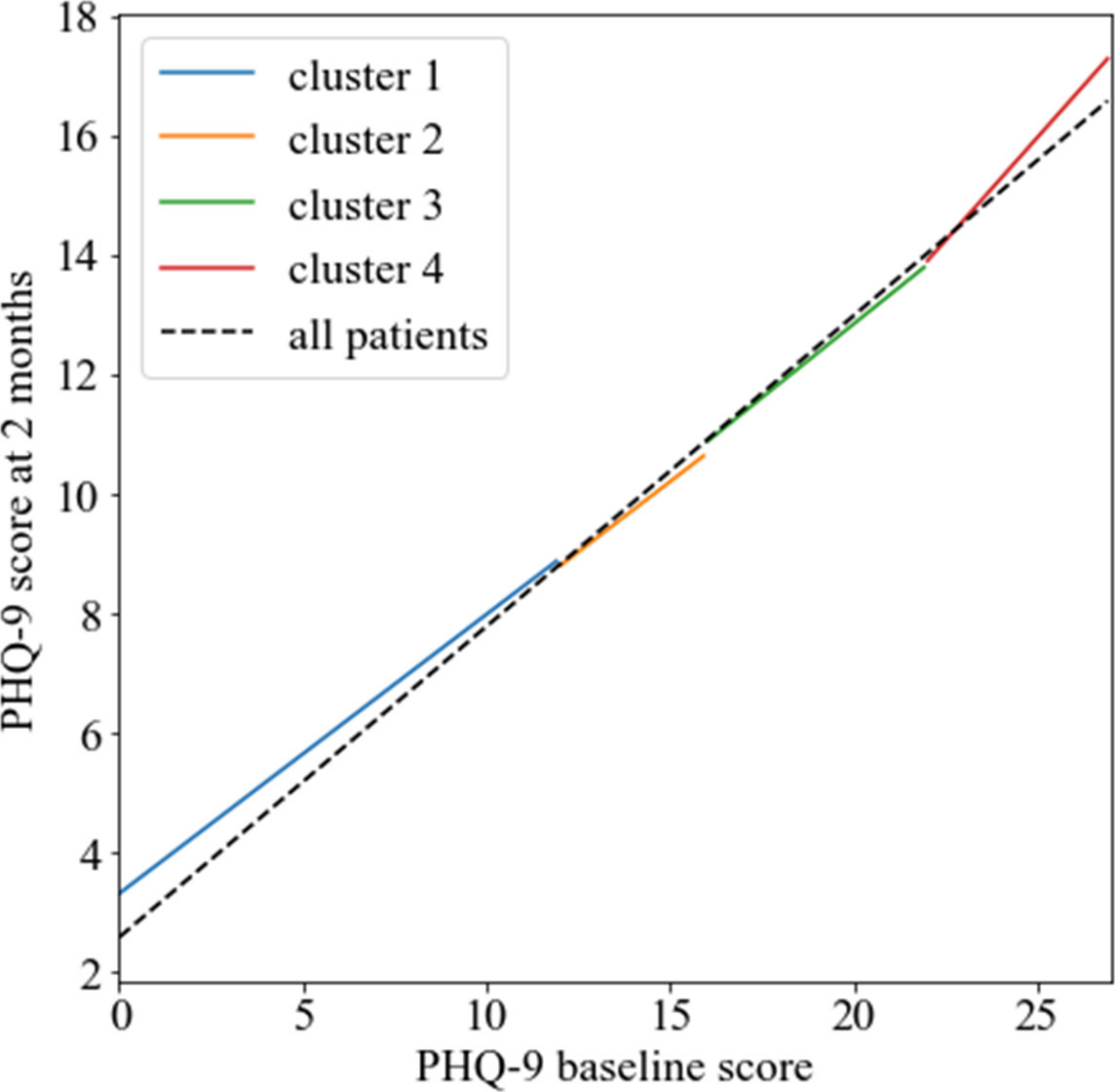
Linear regression models fitted within each of the four clusters. The coloured lines depict the linear regression models fitted within each of the four clusters, that is, the baseline Patient Health Questionnaire (PHQ-9) score versus the score at 2 months as predicted from the models. The dashed line shows predictions from the end-to-end model, fitted in all patients.

We then extracted the coefficients (averaged across 10 imputed data sets) of the most important predictor, PHQ-9 baseline score,^18^ from the regression models developed on the four clusters and all patients, respectively. We found evidence of an overall linear relationship between the outcome and PHQ-9 baseline score of each individual model (figure 3), which also explains the similar performance of the end-to-end and the similarity-based approaches.

The results of the sensitivity analysis, that is, varying the optimal value of *k* from 4 to other values from 2 to 8 and measuring MAE and R^2^ using bootstrapping, are presented in table 2. We found the optimal value of *k* clusters based on the Silhouette coefficient to be consistent with the performances based on the MAE and R^2^ parameters of the similarity-based model. For values of *k* other than 4, the MAE increased and the R^2^ values dropped.

## Discussion

In this paper, we compared the conventional end-to-end approach with a two-stage similarity-based predictive modelling approach with respect to their ability to predict PHQ-9 depression scores using a UK real-world observational data set including 16 384 primary care patients taking fluoxetine. We found that the end-to-end and similarity-based approaches showed comparably low predictive performance for any number of clusters *k* ranging from 2 to 8. This finding is in contrast with previous studies,^8–10^ where the similarity-based approach led to performance benefits. One possible explanation is that the available clinical and demographic variables were not strong predictors of changes in PHQ-9 scores in our data set. The slopes of the regression models were almost identical across the four clusters in the similarity-based approach and similar to the regression model fitted in the entire data set in the end-to-end approach (see figure 3, using the strongest predictor, PHQ-9 baseline score, as an example). This linearity was also seen in a previous publication, where using the same data we found that the linear regression model performed almost identically to more complex non-linear models (ie, other machine learning and deep neural network models).^18^ As the post-treatment PHQ-9 scores were mostly linearly dependent on the predictors, the end-to-end ridge regression model used the full potential of the data set and the similarity-based approach did not lead to any increase in performance. Moreover, forcing the whole data set into smaller clusters resulted in a decrease in sample size for each cluster-specific model and therefore a loss in statistical power for the similarity-based approach.^27^

In theory, an advantage of the similarity-based approach is that clustering results may potentially reveal clinically meaningful patterns among patients.^8^ In our example, the algorithm identified four clusters using seven patient characteristics. These characteristics had consistently increased or decreased the mean values or percentages across the identified clusters. Overall, cluster 1 seemed to group patients with poorer psychosocial functioning compared with other clusters: patients of that group tended to have more severe depressive symptoms at baseline (higher mean PHQ-9 score, at 15.4), tended to be younger (mean age at 42.2), were more likely to be smokers (38.0%), were less likely to be white (90.6%) and were at a higher level of socioeconomic deprivation compared with patients in other clusters (Townsend deprivation score of 1 at 28.3%). These clustering results suggest important correlations between predictors, such as a link between deprivation and psychiatric disorders.

This paper has some potential limitations. First, among several possible prediction models, we opted for one specific model (ie, ridge regression), and in theory using different models could lead to different results. However, we deemed this highly unlikely as we previously compared neural networks with ridge regression for the same data set and found almost identical predictive performance.^18^ Second, our analysis focused only on pharmacological treatments for depression rather than other non-pharmacological treatments such as psychotherapy, which may limit the generalisability of findings as the prediction of non-pharmacological treatment outcomes might require a completely different set of predictors and thus some strong predictors might be found.^28^ Finally, we did not consider neuroimaging and genomic multimodal data in our analyses, which could potentially introduce non-linearities, enlarge the separation between clusters and benefit the predictive performance of the similarity-based approach.^29 30^ The performance of complex models such as deep neural networks should be further assessed in future studies using variables from different international data sets and across other mental health disorders and treatments.

In conclusion, the end-to-end method and two-stage similarity-based modelling approach yielded comparable results when predicting the individual depression symptom severity at 2 months after initiating antidepressant treatment. When limited to demographic and clinical variables as predictors of depression severity, the end-to-end approach can be applied by default because it is easier to perform and we found no evidence of superior performance compared with the similarity-based approach.
