# Supplementary material for: Predicting outcomes at the individual patient level: what is the best method?
Source: BMJ Ment Health. 2023 Jun 14;26(1):e300701. doi: 10.1136/bmjment-2023-300701 (PMC10277128; doi:10.1136/bmjment-2023-300701)
Supplement: Supplementary data [file bmjment-2023-300701supp001.pdf]

Predicting outcomes at the individual patient level: what is the best method? – supplementary materials

Contents

Cohort selection 2

Predictors 5

Hyper-parameter tuning of the ridge regression 6

K-means clustering 7

Silhouette coefficient 8

Exploring patterns in the identified clusters 9

Mock dataset (Table S1) 10

Visualisation of model predictions (Figure S1) 11

Optimal number of clusters (Table S3) 14

Summarised patient characteristics of clusters (Table S4) 15

References 17

Cohort selection

Inclusion criteria

We included patients registered with QResearch with a recorded diagnosis of depression since 1st Jan 1998. As in other studies,[1-3] we used Read codes to identify cases of depression. A new Read code diagnosis of depression was considered a new episode of depression when preceded by 12 months of no depression diagnoses and no prescription of antidepressants. The index date for entry to the cohort was the date of depression diagnosis and patients were followed up for 3 months after the index date.

We considered 12 months of no diagnosis and no prescription of antidepressants as necessary to consider an episode of depression different from the eventual previous one (see below, in the exclusion criteria). This is because with treatment, episodes last on average 3 to 6 months, while most patients recover within 12 months[4] and in general practices the long-term course of depression is more favourable than in clinical samples.[5]

Episodes of depression were included only if fluoxetine was prescribed within 12 days around the diagnosis of depression (i.e. 6 days before or after the diagnosis), because we were interested in the prognosis of patients treated with fluoxetine. Episodes where antidepressants were started 6 or more days prior to the index date of depression diagnosis were considered as if antidepressants were prescribed for other reasons than depression, and were excluded. Episodes with antidepressants prescribed after 6 days were considered as if patients were being on a watchful waiting/active monitoring and were excluded.[6-8]

We did not use a specific threshold on a depression scale (e.g. PHQ-9 >5 at baseline) to include participants, as patients in primary care can be treated even with minor symptoms of

depression[9] and GP ratings of severity do not always agree with validated screening instruments.[10]

Exclusion criteria

We excluded:

- Episodes of depression which had a previous episode of depression in the year before or a previous prescription of antidepressants in the year before. This is because patients with multiple diagnoses and treatments within the year can be treatment-resistant and would therefore be a different population.[11]
- Episodes of depression not associated with a prescription of an antidepressant at baseline (i.e., 6 days before or after the diagnosis).
- Episodes of depression associated with a prescription of more than one antidepressant in the year before (i.e. 365 days before or 6 days after the diagnosis);
- Episodes of depression associated with a prescription of antipsychotics in the year before (i.e. 365 days before or 6 days after the diagnosis);
- Episodes of depression associated with a prescription of mood stabilisers in the year before (i.e. 365 days before or 6 days after the diagnosis);
- Episodes of depression starting within 3 months of delivery (i.e. post-partum depression).

If a patient had multiple episodes of depression recorded, we gave preference to the last episode for each patient.

Liu Q, *et al. BMJ Ment Health* 2023; 26:e300701. doi: 10.1136/bmjment-2023-300701

We also excluded patients with a recorded diagnosis of bipolar disorder or schizophrenia spectrum disorder made at any point before the index episode.

Exposure

The primary exposure of interest was the use of fluoxetine. Information was extracted from all prescriptions for fluoxetine issued during the 3-months follow-up. We calculated the duration of each prescription in days by dividing the number of tablets prescribed by the number of tablets to be taken each day. If the information on tablets per day was missing or not sufficiently detailed (expected to be < 5% of total prescriptions) we estimated the duration of the prescription based on the number of tablets prescribed, as in previous studies.[3] Patients were classified as continually exposed to fluoxetine during periods where there were no gaps of more than 30 days between the end of one prescription and the start of the next (most antidepressants at the beginning of a treatment are prescribed for not more than 28-30 days). Patients were classified as exposed for the first 30 days after the estimated date of stopping fluoxetine in order to account for any delays in starting the prescription or accumulation of tablets as well as to attribute the outcomes occurring during withdrawal periods to the antidepressant, as done in previous studies.

Predictors

Demographic variables included age at diagnosis (continuous), sex (male or female), Townsend quintiles (from 1: least deprived to 5: most deprived), ethnicity (White/Caucasian, African/Caribbean, Asian, Other), Body Mass Index (BMI, continuous), smoking status (currently non-smoker, smoker).

Condition-specific variables included baseline depression severity (continuous, we considered PHQ-9 recorded up to two weeks before and 6 days after the index diagnosis of depression as the baseline measurement), previous antidepressant use (yes/no), use of selective serotonin reuptake inhibitor (SSRI) in the past (yes/no), use of fluoxetine in the past (yes/no), previous psychotherapy use (yes/no), previous referral to secondary care (yes/no), childhood maltreatment (yes/no). Note that the use of medications would be more than a year before, exactly. Episodes where patients were prescribed antidepressants in the year before were excluded.

Comorbid conditions included coronary heart disease, stroke/transient ischemic attack, diabetes, epilepsy/seizures, hypothyroidism, chronic inflammatory diseases (including Osteoarthritis and Rheumatoid arthritis), anxiety or obsessive-compulsive disorder, migraine.

Use of other drugs at baseline included antihypertensive drugs, aspirin, statins, anticoagulants, non-steroidal anti-inflammatory drugs, anticonvulsants, hypnotics/anxiolytics, bisphosphonates, oral contraceptives or hormone replacement therapy.

Liu Q, *et al. BMJ Ment Health* 2023; 26:e300701. doi: 10.1136/bmjment-2023-300701

Hyper-parameter tuning of the ridge regression

To tune the regularisation strength hyper-parameter of ridge regression, in other words, to find the best configuration of ridge regression and ensure its performance against unseen data, a grid search from 0.0001 to 100 was conducted. Specifically, for each of the hyper-parameter candidate values, we performed 10-fold cross validation on the training data, where we first randomly split the training data into 10 folds. We then took 9 folds to train the ridge regression and tested it on the left-out fold. We looped through all 10 folds and in the end, checked the performance of all models. The hyper-parameter that yielded the best performance was selected to develop the ridge regression on all training samples.

Liu Q, *et al. BMJ Ment Health* 2023; 26:e300701. doi: 10.1136/bmjment-2023-300701

K-means clustering

K-means is a distance-based clustering algorithm, which first identifies pre-determined *k* number of centroids (centre of cluster) within an unlabelled multidimensional dataset and then allocates every data point (here a multidimensional data point represents a patient with 31 baseline characteristics) to the nearest cluster, while keeping the centroids as small as possible by reducing the in-cluster sum of squares.

Silhouette coefficient

The Silhouette coefficient is a measure of how similar data points within a cluster are, compared to data points in other clusters. It is calculated as $\frac{(b-a)}{\max(a,b)}$, where a is the mean intra-cluster distance and b is the mean nearest-cluster distance, i.e., the mean distance to the nearest cluster that a sample is not a part of. Of note, “distance” in this context is defined as in the k-means algorithm.

Exploring patterns in the identified clusters

After fitting the similarity-based approach, we examined the identified clusters, to explore patterns among patients therein. For each multiple imputed datasets and for each identified cluster, we summarised the baseline characteristics (which were used for the clustering) as well as the outcomes (not involved in clustering). We did not expect clusters to be identical across the 10 multiple imputed datasets, and in fact, the optimal number of clusters may be different across imputed datasets. However, we hypothesised that the patients and their characteristics in each cluster were similar enough across imputed datasets, with the clustering procedure not being very sensitive to the multiple imputation procedure.

Liu Q, *et al. BMJ Ment Health* 2023; 26:e300701. doi: 10.1136/bmjment-2023-300701

Mock dataset (Table S1)

Table S1. Mock dataset of five patient.

**Predictor/Outcome**	**Patient 1**	**Patient 2**	**Patient 3**	**Patient 4**	**Patient 5**
Sex	Female	Male	Female	Male	Female
Age	43	59	71	26	34
Body Mass Index (BMI)	30.23	32.89	27.71	21.90	19.31
Smoking	Yes	No	No	No	No
Ethnic group	White	Asian	White	White	Other
Townsend deprivation score	3	4	2	5	1
Baseline (PHQ-9)	23	22	19	22	26
First episode	Yes	Yes	Yes	No	No
Use of antidepressant	No	No	No	Yes	No
Use of SSRI	No	No	No	Yes	No
Use of fluoxetine	No	No	No	No	No
Previous psychotherapy	No	No	No	No	Yes
Previous referral to MH care	No	No	No	Yes	No
Childhood maltreatment	No	No	Yes	No	No
Coronary heart disease	No	No	No	No	Yes
Stroke	No	No	Yes	No	No
Diabetes	No	No	Yes	No	No
Epilepsy	No	Yes	No	No	No
Hypothyroidism	No	No	No	No	Yes
Arthritis	No	No	No	No	Yes
Anxiety	No	No	Yes	No	No
Migraine	Yes	No	No	No	No
Antihypertensive	No	No	Yes	No	No
Aspirin	Yes	No	No	No	No
Statins	No	No	No	No	No
Anticoagulants	Yes	No	No	No	No
NSAIDs	No	No	No	No	No
Anticonvulsants	No	Yes	No	No	No
Hypnotics	No	No	No	Yes	No
Bisphosphonates	No	No	No	No	No
Contraceptives	No	No	No	No	Yes
PHQ-9 at 2 months	12	20	11	6	9

Visualisation of model predictions (Figure S1)

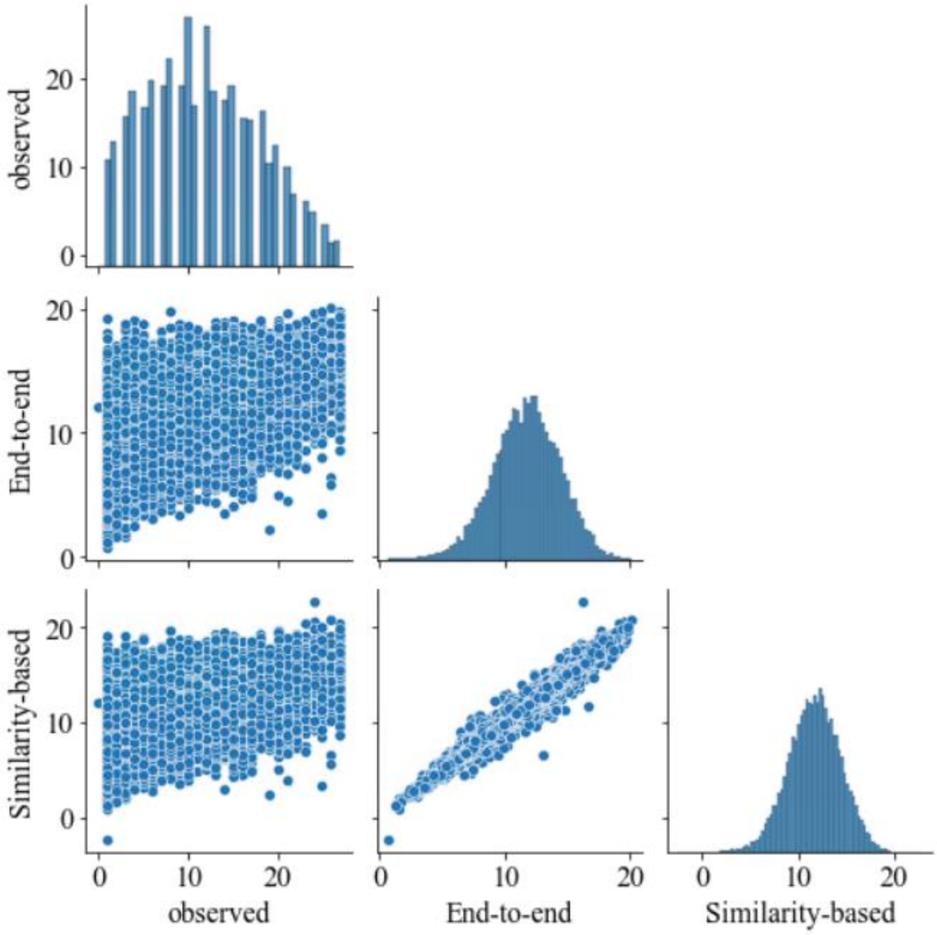

Figure S1. Paired plots of the predicted Patient Health Questionnaire (PHQ-9) scores of the end-to-end and similarity-based models (k = 4).

Liu Q, *et al. BMJ Ment Health* 2023; 26:e300701. doi: 10.1136/bmjment-2023-300701

Table S2. Average coefficients of the ridge end-to-end models developed on 10 imputed datasets.

**Predictor**	**Coefficient**
Sex (male or female)	1.19×10^{-2}
First episode	6.26×10^{-4}
Fluoxetine used in the past	-3.79×10^{-3}
Selective serotonin reuptake inhibitor (SSRI) used in the past	-3.88×10^{-3}
Antidepressant used in the past	1.46×10^{-2}
Body mass index	9.23×10^{-3}
Currently smoker or non-smoker	1.61×10^{-2}
Patient Health Questionnaire (PHQ-9) baseline depression severity	5.13×10^{-1}
Previous psychotherapy use	1.36×10^{-4}
Previous referral to secondary care	-8.53×10^{-4}
Childhood maltreatment	-6.20×10^{-4}
Comorbid condition - coronary heart disease	2.07×10^{-3}
Comorbid condition - stroke/transient ischaemic attack	2.15×10^{-3}
Comorbid condition - diabetes	1.17×10^{-3}
Comorbid condition - epilepsy/seizures	-9.49×10^{-4}
Comorbid condition - hypothyroidism	-1.53×10^{-3}
Comorbid condition - chronic inflammatory diseases (including Osteoarthritis and Rheumatoid arthritis)	-1.96×10^{-3}
Comorbid condition - anxiety or obsessive-compulsive disorder	3.75×10^{-3}
Comorbid condition - migraine	5.76×10^{-4}
Use of antihypertensive drugs at baseline	-5.11×10^{-4}
Use of aspirin at baseline	9.66×10^{-4}
Use of statins at baseline	-2.41×10^{-3}
Use of anticoagulants at baseline	-2.74×10^{-4}
Use of non-steroidal anti-inflammatory drugs at baseline	6.35×10^{-3}
Use of anticonvulsants at baseline	3.86×10^{-3}
Use of hypnotics/anxiolytics at baseline	2.35×10^{-3}
Use of bisphosphonates at baseline	-1.50×10^{-3}
Use of oral contraceptives or hormone replacement therapy at baseline	-4.86×10^{-3}
Age at diagnosis	-1.25×10^{-2}
Ethnicity (white/Caucasian)	-6.90×10^{-3}
Ethnicity (African/Caribbean)	5.00×10^{-3}
Ethnicity (Asian)	5.74×10^{-3}

Ethnicity (Other)	8.63×10^{-4}
Townsend quintile score 1	-1.13×10^{-2}
Townsend quintile score 2	-7.58×10^{-3}
Townsend quintile score 3	7.46×10^{-4}
Townsend quintile score 4	8.66×10^{-3}
Townsend quintile score 5	1.31×10^{-2}

Liu Q, *et al. BMJ Ment Health* 2023; 26:e300701. doi: 10.1136/bmjment-2023-300701

Optimal number of clusters (Table S3)

Table S3. Values of the Silhouette coefficient calculated by running *k*-means clustering on 1,000 bootstrapped samples.

Clusters (Number)	Values of the Silhouette coefficient [95% CI[1]]
2	0.142 [0.137, 0.146]
3	0.141 [0.138, 0.145]
4	0.148 [0.121, 0.157]
5	0.138 [0.070, 0.167]
6	0.125 [0.078, 0.174]
7	0.117 [0.082, 0.176]
8	0.114 [0.089, 0.172]

[1] Confidence interval, calculated as the 2.5th to the 97.5th percentile of bootstrap estimates.

Liu Q, *et al. BMJ Ment Health* 2023; 26:e300701. doi: 10.1136/bmjment-2023-300701

Summarised patient characteristics of clusters (Table S4)

Table S4. Summarised patient characteristics of clusters based on the optimal cluster number $k = 4$.

**Cluster ID**	**1**	**2**	**3**	**4**
Number of patients	3390	6514	4794	1686
PHQ-9 baseline score (mean, standard deviation)	23.79 [1.55]	18.99 [1.41]	14.21 [1.38]	8.65 [2.50]
PHQ-9 at 2 months (mean, standard deviation)	15.41 [6.61]	12.41 [5.95]	9.71 [5.32]	7.16 [4.85]
Difference of the PHQ-9 scores between baseline and at 2 months (mean, standard deviation)	8.38 [6.52]	6.58 [5.94]	4.50 [5.33]	1.49 [4.89]
Male	38.4%	37.8%	36.3%	39.0%
First episode	67.8%	67.6%	66.4%	67.2%
Fluoxetine used in the past	22.1%	22.5%	24.3%	23.8%
Selective serotonin reuptake inhibitor (SSRI) used in the past	31.5%	31.4%	32.3%	31.7%
Antidepressant used in the past	37.9%	38.1%	39.0%	38.7%
Body mass index	27.4%	27.2%	27.0%	26.9%
Currently smoker	38.0%	31.9%	27.1%	22.1%
Previous psychotherapy use	1.1%	0.9%	1.0%	0.7%
Previous referral to secondary care	0.3%	0.3%	0.4%	0.5%
Childhood maltreatment	0.2%	0.1%	0.2%	0.1%
Comorbid condition - coronary heart disease	2.4%	2.4%	2.5%	3.9%
Comorbid condition - stroke/transient ischaemic attack	1.4%	1.5%	1.6%	2.0%
Comorbid condition - diabetes	5.0%	5.1%	4.7%	6.1%
Comorbid condition - epilepsy/seizures	1.1%	1.2%	1.2%	0.8%
Comorbid condition - hypothyroidism	3.8%	3.8%	3.9%	3.5%
Comorbid condition - chronic inflammatory diseases (including Osteoarthritis and Rheumatoid arthritis)	6.4%	7.0%	7.2%	9.8%
Comorbid condition - anxiety or Obsessive-compulsive disorder	17.2%	15.8%	15.1%	16.8%
Comorbid condition - migraine	9.3%	9.7%	9.2%	8.5%
Use of antihypertensive drugs at baseline	7.6%	8.6%	8.7%	11.2%
Use of aspirin at baseline	3.1%	3.5%	3.6%	5.3%
Use of statins at baseline	7.5%	7.7%	8.0%	11.2%

Use of anticoagulants at baseline	0.4%	0.6%	0.7%	0.9%
Use of non-steroidal anti-inflammatory drugs at baseline	4.8%	3.9%	3.5%	2.8%
Use of anticonvulsants at baseline	1.6%	1.1%	1.4%	1.2%
Use of hypnotics/anxiolytics at baseline	9.9%	7.8%	5.8%	5.6%
Use of bisphosphonates at baseline	0.3%	0.2%	0.3%	0.5%
Use of oral contraceptives or hormone replacement therapy at baseline	5.8%	6.6%	6.6%	5.0%
Age at diagnosis	42.17	42.81	44.27	47.76
Ethnicity (white)	90.6%	92.8%	93.5%	93.9%
Ethnicity (African)	1.9%	1.7%	1.0%	1.2%
Ethnicity (Asian)	4.3%	3.6%	3.6%	2.8%
Ethnicity (other)	3.2%	2.4%	1.8%	2.1%
Townsend deprivation score 1	19.7%	22.5%	25.3%	28.3%
Townsend deprivation score 2	21.9%	23.6%	26.1%	24.3%
Townsend deprivation score 3	22.6%	22.1%	20.7%	20.6%
Townsend deprivation score 4	20.4%	18.1%	16.8%	15.5%
Townsend deprivation score 5	15.4%	13.7%	11.2%	11.3%

Liu Q, *et al. BMJ Ment Health* 2023; 26:e300701. doi: 10.1136/bmjment-2023-300701

References

1. De Crescenzo F, Garriga C, Tomlinson A, et al. Real-world effect of antidepressants for depressive disorder in primary care: protocol of a population-based cohort study. *Evidence-Based Mental Health* 2020;23(3):122-26.

2. Coupland C, Dhiman P, Morriss R, et al. Antidepressant use and risk of adverse outcomes in older people: population based cohort study. *BMJ* 2011;343

3. Coupland C, Hill T, Morriss R, et al. Antidepressant use and risk of adverse outcomes in people aged 20–64 years: cohort study using a primary care database. *BMC Medicine* 2018;16:1-24.

4. Malhi G, Mann J. Depression. *Lancet* 2018;10161(392):2299–312.

5. Steinert C, Hofmann M, Kruse J, et al. The prospective long-term course of adult depression in general practice and the community. A systematic literature review. *Journal of Affective Disorders* 2014;152:65-75.

6. Hegel MT, Oxman TE, Hull JG, et al. Watchful waiting for minor depression in primary care: remission rates and predictors of improvement. *Gen Hosp Psychiatry* 2006;28:205-12.

7. Moore M, Byng R, Stuart B, et al. 'Watchful waiting' or 'active monitoring' in depression management in primary care: Exploring the recalled content of general practitioner consultations. *Journal of Affective Disorders* 2013;145(1):120-25.

8. Iglesias-González M, Aznar-Lou I, Gil-Girbau M, et al. Comparing watchful waiting with antidepressants for the management of subclinical depression symptoms to mild–moderate depression in primary care: a systematic review. *Family Practice* 2017;34(6):639-48.

9. Barley EA, Murray J, Walters P, et al. Managing depression in primary care: a meta-synthesis of qualitative and quantitative research from the UK to identify barriers and facilitators. *BMC Family Practice* 2011;12(1):1-11.

10. Kendrick T, King F, Albertella L, et al. GP treatment decisions for patients with depression: an observational study. *British Journal of General Practice* 2005;55:280-86.

11. Wiles N, Taylor A, Turner N, et al. Management of treatment-resistant depression in primary care: a mixed-methods study. *British Journal of General Practice* 2018;68(675):e673-e81.
